# End-expiratory occlusion maneuver to predict fluid responsiveness in the intensive care unit: an echocardiographic study

**DOI:** 10.1186/s13054-017-1938-0

**Published:** 2018-02-08

**Authors:** Delphine Georges, Hugues de Courson, Romain Lanchon, Musa Sesay, Karine Nouette-Gaulain, Matthieu Biais

**Affiliations:** 1 Department of Anesthesiology and Critical Care Pellegrin, Bordeaux University Hospital, F-33000 Bordeaux, France; 2 INSERM, U12-11, Laboratoire de Maladies Rares: Génétique et Métabolisme (MRGM), Bordeaux, France; 3 University of Bordeaux, Bordeaux, F-33600 France; 4 INSERM, U1034, Biology of Cardiovascular Diseases, F-33600 Pessac, France

**Keywords:** Fluid responsiveness, End-expiratory occlusion, Heart-lung interactions, Volume expansion, Echocardiography

## Abstract

**Background:**

In mechanically ventilated patients, an increase in cardiac index during an end-expiratory-occlusion test predicts fluid responsiveness. To identify this rapid increase in cardiac index, continuous and instantaneous cardiac index monitoring is necessary, decreasing its feasibility at the bedside. Our study was designed to investigate whether changes in velocity time integral and in peak velocity obtained using transthoracic echocardiography during an end-expiratory-occlusion maneuver could predict fluid responsiveness.

**Methods:**

This single-center, prospective study included 50 mechanically ventilated critically ill patients. Velocity time integral and peak velocity were assessed using transthoracic echocardiography before and at the end of a 12-sec end-expiratory-occlusion maneuver. A third set of measurements was performed after volume expansion (500 mL of saline 0.9% given over 15 minutes). Patients were considered as responders if cardiac output increased by 15% or more after volume expansion.

**Results:**

Twenty-eight patients were responders. At baseline, heart rate, mean arterial pressure, cardiac output, velocity time integral and peak velocity were similar between responders and non-responders. End-expiratory-occlusion maneuver induced a significant increase in velocity time integral both in responders and non-responders, and a significant increase in peak velocity only in responders. A 9% increase in velocity time integral induced by the end-expiratory-occlusion maneuver predicted fluid responsiveness with sensitivity of 89% (95% CI 72% to 98%) and specificity of 95% (95% CI 77% to 100%). An 8.5% increase in peak velocity induced by the end-expiratory-occlusion maneuver predicted fluid responsiveness with sensitivity of 64% (95% CI 44% to 81%) and specificity of 77% (95% CI 55% to 92%). The area under the receiver operating curve generated for changes in velocity time integral was significantly higher than the one generated for changes in peak velocity (0.96 ± 0.03 versus 0.70 ± 0.07, respectively, *P* = 0.0004 for both). The gray zone ranged between 6 and 10% (20% of the patients) for changes in velocity time integral and between 1 and 13% (62% of the patients) for changes in peak velocity.

**Conclusions:**

In mechanically ventilated and sedated patients in the neuro Intensive Care Unit, changes in velocity time integral during a 12-sec end-expiratory-occlusion maneuver were able to predict fluid responsiveness and perform better than changes in peak velocity.

## Background

Management of fluid administration is of major importance in the intensive care unit (ICU) and particularly in the neuro-ICU. On the one hand, hypovolemia may lead to organ dysfunction and on the other hand positive cumulative fluid balance is associated with an increase in both morbidity and mortality [1]. Recent studies underline the heterogeneity of practice and the uncommon prediction of fluid responsiveness before volume expansion [2, 3]. Dynamic parameters such as pulse pressure variations and stroke volume variations are very robust parameters but many limitations have been described in the ICU [4–6]. New approaches (evaluation of the effects of a transient increase in tidal volume, of a lung recruitment maneuver or an end-expiratory occlusion (EEO) test) have been developed [7–9].

Monnet et al. were the first to investigate the possibility of predicting fluid responsiveness by analyzing the effects of an EEO [8]. In patients under mechanical ventilation, the inspiratory phase increases intra-thoracic pressure and decreases venous return. EEO prevents any variation in intra-thoracic pressure. This leads to an increase in venous return, cardiac preload and stroke volume in preload-responsive patients. Thus, an increase in cardiac index during an EEO could predict fluid responsiveness. In order to identify the rapid and transient increase in cardiac index during the EEO, continuous and instantaneous cardiac index monitoring is necessary (pulse contour analysis was used in ICU studies).

Transthoracic echocardiography is routinely used in the ICU and may allow continuous measurements of stroke volume and cardiac output [10]. In patients who do not benefit from continuous cardiac output monitoring, echocardiography could be an interesting alternative to track changes in stroke volume or cardiac output [11]. The aim of the present study was to investigate whether changes in velocity time integral (VTI) and peak velocity (Vmax) during an EEO could predict fluid responsiveness in mechanically ventilated ICU patients.

## Methods

### Patients

The present study was approved by the Institutional Review Board (Comité de Protection des Personnes Sud-Ouest et Outre Mer III, Bordeaux, France number DC2016/14). Fifty non-consecutive patients were prospectively included after informed consent from the patient’s next of kin. Inclusion criteria were sedated and mechanically ventilated patients for whom the decision to perform volume expansion was taken by the physician (arterial hypotension, oliguria less than 0.5 ml/kg/h, skin mottling, attempt to decrease vasopressor infusion rate). Patients were not included if they were younger than 18 years, presented with unsatisfactory cardiac echogenicity, chronic arrhythmia, significant valvular heart disease, intracardiac shunt, left ventricular ejection fraction <50%, right ventricular dysfunction (attested by a peak systolic velocity of tricuspid annular motion <0.15 m/s) or intra-cranial hypertension or spontaneous breathing activity.

### Hemodynamic monitoring

All echocardiographic measurements were performed by two experienced physicians (level 2 or 3) who were unaware of the clinical data (DG and MB), using a General Electric Vivid S6 machine (GE Healthcare, Wauwatosa, WI, USA). Videos were anonymously recorded for blinded and offline measurements, by a single observer (DG). Using the 5-chamber apical view, the VTI was measured from the area under the envelope of the pulsed-wave Doppler signal obtained at the level of the aortic annulus. The VTI and Vmax values were averaged over five consecutive measurements and were manually traced. Using the parasternal long axis view, The diameter of the aortic cusp was measured during systolic time, using the parasternal long axis view. Aortic valve area was calculated as follow: (π diameter ^2^/4). Stroke volume was calculated as the product of VTI by aortic valve area. Cardiac output was calculated as the product by stroke volume and heart rate. Using the apical 2-chamber and 4-chamber views, Simpson’s biplane was used to measure left ventricular ejection fraction.

The reproducibility of VTI and Vmax were assessed before the study. To limit the effects of respiratory-induced changes in VTI and Vmax, each VTI and Vmax value was obtained as an average over five consecutive measurements. VTI and Vmax values were obtained twice in ten patients by the same operator (DG; intra-observer reproducibility) and a second observer (MB; inter-observer reproducibility). The absolute mean difference was calculated and divided by the mean of the two values. Intra-observer variability was 5 ± 1% for VTI and 6 ± 2% for Vmax. Inter-observer variability was 4 ± 2% for VTI and 6 ± 2% for Vmax. We calculated the last significant changes as previously described [12]. Briefly, the coefficient of variation may be assimilated to intra-observer variability. The coefficient of error was calculated as the coefficient of variation divided by √n (*n* = number of replicates of measurements in each patient). Finally, the least significant change was calculated as:

$$ \mathrm{Coefficient}\ \mathrm{of}\ \mathrm{error}\times 1.96\times \surd 2 $$

The least significant change was 9.8% for VTI and 11.8% for Vmax.

### Respiratory parameters

Patients were ventilated in the volume control mode (Servo-U, Maquet Medical System, Wayne, NJ, USA). Tidal volume was set between 6 and 8 mL/kg of ideal body weight. End-expiratory occlusion has been extensively described elsewhere. Briefly, it was performed by interrupting the ventilator at end-expiration over 12 sec using the automatic and specific device of the ventilator (total positive end-expiratory pressure). An investigator observed the curves displayed on the ventilator to ensure the absence of spontaneous breathing effort during the end-expiratory occlusion maneuver.

### Study design

All patients were in supine position (trunk elevated 30°), sedated and mechanically ventilated. Three sets of measurements were performed. The first set was baseline and included heart rate, arterial pressure, left ventricular ejection fraction, diameter of the aortic cusp, VTI and Vmax measurements. We considered the diameter of the aortic cusp constant during the study protocol. The second set was performed at the end of a 12-sec end-expiratory occlusion maneuver. At this time, heart rate, arterial pressure, VTI and Vmax measurements were recorded (the measurement were performed on the last five cycles before the end of EEO). After the end of the end-expiratory occlusion maneuver, volume expansion was performed using 500 mL of saline 0.9% over 15 minutes. A third set of measurements was performed immediately after the end of the fluid administration. This set included heart rate, arterial pressure and VTI and Vmax measurements. The VTI and Vmax values were averaged over five consecutive measurements.

### Statistical analysis

Normality was tested using the d’Agostino-Pearson test. Data were expressed as median (25th–75th percentile) or mean (standard derivation, SD) as appropriate. Differences between hemodynamic variables were evaluated with the Wilcoxon test or *t* test as appropriate. Response to volume expansion was defined as an increase in cardiac output of 15% or more [4, 13]. Receiver operating characteristic (ROC) curves were generated to evaluate the ability of percentage changes in VTI and Vmax induced by a 12-sec end-expiratory occlusion maneuver to predict a fluid-induced increase in cardiac output ≥15%. The ROC curves were compared using the DeLong test [14]. The best threshold value was chosen so as to maximize the Youden Index (specificity + sensitivity – 1). In order to avoid the binary response provided by the ROC curves and to take into account an overlap between responders and non-responders, a gray zone was determined for changes in VTI and changes in Vmax. The gray zone was constructed using a two-step procedure. First, a bootstrap resampling method was applied on changes in VTI and Vmax data. The best threshold of 1000 bootstrapped populations and its 95% CI were chosen for each variable. Second, we determined the values for which no conclusive information on fluid responsiveness (i.e., cutoff values with sensitivity <90% or specificity <90% (diagnostic tolerance of 10%) could be provided. The gray zone was defined as the values that did not allow a 10% diagnostic tolerance. Nevertheless, if the characteristics of the study population produced a 95% CI of the best thresholds larger than the inconclusive zone, the values obtained during the first step were retained as the gray zone. A diagnostic test is considered to have good accuracy when its area under the ROC curve is ≥ 0.75 [15]. Fifty patients were necessary to demonstrate the ability of EEO to predict fluid responsiveness with good accuracy, i.e., area under the ROC curve is ≥ 0.75 (type I error of 5% and type II error of 10%).

Statistical analysis was performed using MedCalc (Version 14.12.0, MedCalc Software bvba, Belgium). A *P* value <0.05 was used for statistical significance.

## Results

### Patients

Fifty non-consecutive patients were included. The main characteristics of patients are reported in Table 1. The main etiology of ICU admission was subarachnoid hemorrhage. A large majority (94%) of patients received norepinephrine and none of them received other vasopressors or inotropes. Left ventricular ejection fraction was higher than 50% in both responders and non-responders.

**Table 1:** Main characteristics of patients

Characteristics	Responders *n* = 28	Non-responders *n* = 22
Age (years)	54 ± 13	53 ± 14
Sex, male/female (*n*)	16/12	10/12
Height (cm)	170 ± 9	169 ± 10
Weight (kg)	77 ± 15	72 ± 17
SAPS II	46 ± 17	46 ± 14
Tidal volume (mL.kg^-1^ of predicted body weight)	6.9 ± 0.7	6.8 ± 0.8
Respiratory rate (breath.min^-1^)	17 ± 5	16 ± 3
Positive end-expiratory pressure (cmH_2_O)	6 ± 2	6 ± 1
Driving pressure (cmH_2_O)	10 ± 3	10 ± 4
Compliance of the respiratory system (mL/cmH_2_O)	49 ± 17	51 ± 18
PaO_2_/FiO_2_	294 ± 125	300 ± 126
Etiology of disease on ICU admission		
Subarachnoid hemorrhage (*n*)	14	12
Intracerebral hemorrhage (*n*)	5	6
Ischemic stroke (*n*)	5	1
Other (*n*)	4	3
Etiology of volume expansion		
Need to increase mean arterial pressure (*n*)	19	12
Oliguria (*n*)	2	7
Tachycardia (*n*)	7	3
Left ventricular ejection fraction (%)	55 ± 9	56 ± 9
Norepinephrine (*n* (%))	25 (89%)	22 (100%)
Dosage of norepinephrine (μg.kg^-1^.min^-1^)	0.49 (0.19–1.5)	0.90 (0.5–1.80)

Values are mean ± SD, number of patients (*n*) or median (interquartile range (25–75%)) as appropriate

*ICU* Intensive Care Unit, *SAPS II* Simplified Acute Physiologic Score, *PaO*_*2*_*/FiO*_*2*_ ratio of arterial oxygen tension to inspired oxygen fraction

### Effects of EEO and volume expansion

EEO induced a significant increase in VTI (19% in responders and 5% in non-responders), in Vmax (10% in responders and in 4% non-responders), in stroke volume (18% in responders and 4% in non-responders) and cardiac output (20% in responders and 6% in non-responders). Volume expansion induced a significant increase in mean arterial pressure, VTI, stroke volume and cardiac output in both responders and non-responders. Volume expansion induced an increase in Vmax only in responders (Table 2).

**Table 2:** Hemodynamic variables at baseline, at the end of the end-expiratory occlusion test and after volume expansion in responders (*n* = 28) and non-responders (*n* = 22)

Variables	Baseline	EEO	After VE	*P*1	*P*2
Heart rate (bpm)					
Responders	71 ± 17	71 ± 16	73 ± 16	0.59	0.006
Non-responders	74 ± 18	74 ± 17	74 ± 18	0.99	0.8
Mean arterial pressure (mmHg)					
Responders	82 ± 15	91 ± 11	94 ± 16	0.34	0.0001
Non-responders	85 ± 12	85 ± 14	96 ± 13	0.18	0.0002
Cardiac output (l/min)					
Responders	5. 1 ± 2.0	6.1 ± 2.8	6.4 ± 2.7	<0.0001	<0.0001.
Non-responders	5.0 ± 1.6	5.3 ± 1.8	5.3 ± 1.6	0.0006	0.006
Stroke volume (mL)					
Responders	72 ± 20	85 ± 29	87 ± 28	<0.0001	<0.0001
Non-responders	70 ± 23	73 ± 23	73 ± 23	0.0012	0.02
Velocity time integral (cm)					
Responders	21 ± 5	25 ± 5	25 ± 5	<0.0001	<0.0001
Non-responders	21 ± 4	22 ± 4	22 ± 5	0.0001	0.01
Vmax (cm/sec)					
Responders	1.13 ± 0.26	1.25 ± 0.30	1.27 ± 0.3	<0.0001	<0.0001
Non-responders	1.10 ± 0.25	1.15 ± 0.24	1.15 ± 0.23	<0.001	<0.05

Values are mean ± standard deviation. Patients were considered responders if stroke volume increased by ≥ 10% after 250 mL intravascular volume expansion. Baseline was before end-expiratory occlusion (EEO). The EEO measurements were made at the end of 12-sec EEO. *After VE* measurements made immediately after volume expansion (VE) (500 ml saline), *P1 P* values for comparison between measurements at baseline and at the end of EEO, *P2 P* values for comparison between measurements at baseline and after volume expansion, *Vmax* peak velocity of aortic blood flow

*P* < 0.05 for comparison of responders and non-responders at baseline

### Prediction of fluid responsiveness

**Fig. 1:**
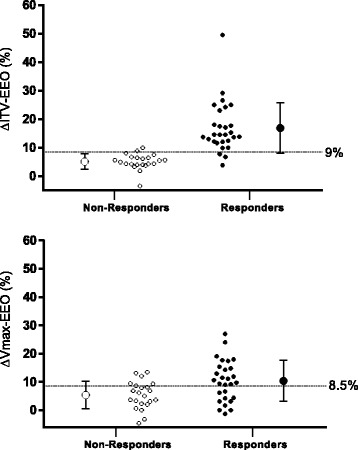
Individual values in responders (*n* = 28) and non-responders (*n* = 22) of the variations in velocity time integral (%) and peak velocity of aortic blood flow (%) during a 12-sec end-expiratory occlusion maneuver. ∆VTI-EEO, changes in velocity time integral (%) induced by end-expiratory occlusion. ∆Vmax-EEO, changes in peak velocity induced by end-expiratory occlusion. Responders, change in cardiac output ≥15% after volume expansion; non-responders, change in cardiac output <15% after volume expansion. Volume expansion, 500 mL saline 0.9% given over 15 minutes

**Fig. 2:**
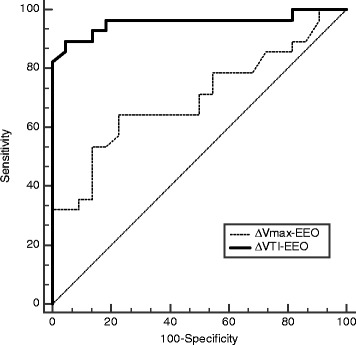
Receiver operating characteristics curves evaluating the ability of the variations in velocity time integral (%) and peak velocity of aortic blood flow (%) during a 12-sec end-expiratory occlusion maneuver to predict fluid responsiveness. ∆VTI-EEO, changes in velocity time integral (%) induced by end-expiratory occlusion. ∆Vmax, changes in peak velocity induced by end-expiratory occlusion. Responders, change in cardiac output ≥15% after volume expansion; non-responders, change in cardiac output <15% after volume expansion. Volume expansion, 500 mL saline 0.9% given over 15 minutes

**Fig. 3:**
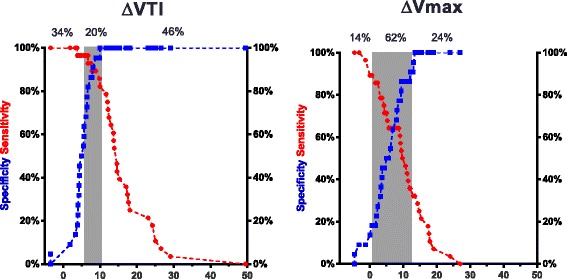
Gray zone of the variations in velocity time integral (%) and peak velocity of aortic blood flow (%) induced by a 12-sec end-expiratory occlusion maneuver to predict fluid responsiveness. The blue curve indicates sensitivity, and the red curve indicates specificity. ∆VTI-EEO, changes in velocity time integral (%) induced by end-expiratory occlusion test. ∆Vmax, changes in peak velocity induced by end-expiratory occlusion. Responders, change in cardiac output ≥15% after volume expansion; non-responders, change in cardiac output <15% after volume expansion. Volume expansion, 500 mL saline 0.9% given over 15 minutes

The main results on the prediction of fluid responsiveness are shown in Figs. 1, 2 and Table 3. A 9% increase in VTI induced by the EEO predicted fluid responsiveness with sensitivity of 89% (95% CI 72% to 98%) and specificity of 95% (95% CI 77% to 100%). An 8.5% increase in peak velocity induced by the EEO maneuver predicted fluid responsiveness with sensitivity of 64% (95% CI 44% to 77%) and specificity of 77% (95% CI 55% to 92%). The area under the ROC curve generated for changes in VTI was significantly higher than the one generated for changes in peak velocity (0.96 ± 0.03 versus 0.70 ± 0.07, respectively, *P* = *P* = 0.0004). The gray zone ranged between 6 and 10% (20% of the patients) for changes in VTI and between 1 and 13% (62% of the patients) for changes in Vmax (Fig. 3).

**Table 3:** Ability to predict increase in cardiac output ≥15% after infusion of 500 mL saline over 15 minutes

Index	Best threshold	Gray zone	Patients whose measurements were in the gray zone	AUROC (95% CI)	Sensitivity (95% CI)	Specificity (95% CI)	Youden index J
ΔVTI	>9%	6–10%	20%	0.96 ± 0.03	89% (72–98%)	95% (77–100%)	0.85
ΔVmax	>8.5%	1–13%	62%	0.70 ± 0.07	64% (44–81%)	77% (55–92%)	0.42

Best threshold value was determined using the Youden index. Youden Index J = Sensitivity + Specificity – 1. ΔVTI represents changes in velocity time integral induced by end-expiratory occlusion. ΔVmax represents changes in peak velocity induced by end-expiratory occlusion

*AUROC* area under receiver operating characteristics curves, *CI* confidence interval

## Discussion

Our study suggests, in mechanically ventilated neuro-ICU patients that: (1) EEO induces an increase in VTI and Vmax; (2) a 9% increase in VTI induced by the end-expiratory-occlusion maneuver predicted fluid responsiveness with satisfactory sensitivity and specificity; and (3) changes in Vmax induced by EEO performed less effectively than changes in VTI and exhibited a larger gray zone.

Monnet et al. were the first to introduce EEO as a marker of fluid responsiveness [8]. Their first study included 34 mechanically ventilated ICU patients and showed that EEO is able to predict fluid responsiveness with satisfactory sensitivity and specificity even in patients with cardiac arrhythmia or moderate spontaneous breathing activity. Later, the same group demonstrated in patients that EEO remains accurate even in patients suffering from acute respiratory distress syndrome and/or low compliance of the respiratory system [16]. More recently, Myatra et al. included 20 patients and suggested that EEO is able to predict fluid responsiveness in patients ventilated with a tidal volume of 8 mL/kg^-1^ of ideal body weight but not in those ventilated with a lower tidal volume (6 mL/kg^-1^ of ideal body weight) [7].

The main difference between these studies and ours is that we used transthoracic echocardiography and not pulse contour analysis for cardiac output measurements. A recent study evaluated the effects of EEO on VTI [17]. Jozwiak et al. included 30 ICU patients and found that a 5% increase in VTI at the end of a 15-sec EEO predicted fluid responsiveness with good sensitivity and specificity. The authors also demonstrated that when combining the effect of EEO and end-inspiratory occlusion on VTI, the threshold discriminating responders and non-responders increased to 13%. This approach is of interest because combining the effects of EEO and end-inspiratory occlusion on VTI allows us to increase the best threshold value (from 5 to 13%) and thus, above the variability in the measurements when using echocardiography. In the present study, we found a higher threshold for both VTI and Vmax (9 and 8.5% respectively). The main difference between these two studies is that the populations are not comparable: different types of patients, hemodynamic baseline values, respiratory parameters, indication of volume expansion, normal compliance of the respiratory system and no acute respiratory distress syndrome in our study, etc. Furthermore, defining thresholds using receiver operating characteristics curves in a small sample needs to take into account the gray zone. Considering VTI, the lower value of the gray zone in our study was very close to best threshold value identified by Jozwiak et al. (6 versus 5%). Other studies including more patients would help us to define more precise thresholds.

Echocardiography is useful for diagnosis and management of acute circulatory failure in ICU patients. It allows rapid assessment of the anatomy and function of the heart and it is possible to directly measure absolute values or changes in stroke volume and cardiac output with good accuracy [11]. Echocardiography may also provide the assessment of both the efficacy and tolerance of fluid challenge. VTI is a major determinant of stroke volume which is calculated as the product of VTI and the aortic valve area. Changes in VTI may be used as a surrogate for changes in stroke volume if we consider that aortic valve area is constant [18, 19]. Some authors propose using changes in Vmax as a surrogate for changes in stroke volume [20].

Echocardiography provides very important information to the clinician and is non-invasive. However, some limitations should be emphasized. First, measurements obtained by echocardiography are dependent on the patients’ echogenicity. ICU patients under mechanical ventilation are well-known for having lower echogenicity. Unfortunately, we did not record in our study the number of patients with poor echogenicity to perform measurements and who had to be excluded. In the recent study published by Jozwiak et al. 41% of patients were excluded because they presented with poor echogenicity. Second, specific training is needed. Many studies demonstrate that learning curves are relatively short for basic measurements (including VTI) [10, 21]. Third, measurements are operator dependent. Intra-observer and inter-observer variability reported in previous studies and in ours is close to 4–6% [17, 22, 23]. This may explain that performance of Vmax was inferior to performance of VTI in the present study. We observed a large gray zone for Vmax (1–13%) with a lower limit lower than the intra-observer and inter-observer variability, whereas this was not the case for VTI (the gray zone ranged between 6 and 10%). Nevertheless, when considering a least significant change just below 10% for VTI measurements in our study, we may have to consider the higher limit of the gray zone (10%) for determining the optimal threshold.

Our study presents some limitations. We did not perform a set of measurements before volume expansion. EEOT is a very quick test (12 sec) and all studies investigating the ability of EEOT to predict fluid responsiveness observed that the effects of EEOT were very transient (<1 minute) and that all hemodynamic parameters returned to their initial values within 1 minute [8, 16, 24]. We included a small sample (*n* = 50) of selected and non-consecutive patients. A large majority of our patients suffered from subarachnoid hemorrhage and volume expansion was done to increase mean arterial pressure to improve cerebral blood flow. Few of them had sepsis or septic shock. Furthermore, our patients did not suffer from acute respiratory distress syndrome, had normal compliance with the respiratory system and were ventilated using low positive end-expiratory pressure levels (6 cmH_2_O). This may decrease the impact of our results and decrease the external validity of our study. Nevertheless, our study was able to show that changes in stroke volume induced by an EEO may be assessed using transthoracic echocardiography and that the magnitude of changes in stroke volume may help to predict fluid responsiveness.

## Conclusion

The present study suggests that in mechanically ventilated and sedated ICU patients, changes in stroke volume induced by a 12-sec EEO may be assessed using transthoracic echocardiography. Change in VTI was able to predict fluid responsiveness and performed better than change in Vmax.

## Abbreviations

CI: Confidence interval
EEO: End-expiratory occlusion
ICU: Intensive care unit
ROC: Receiver operating characteristics
Vmax: Peak velocity of aortic blood flow
VTI: Velocity time integral
